# Design and Fabrication of a MEMS Electromagnetic Swing-Type Actuator for Optical Switch

**DOI:** 10.3390/mi12020221

**Published:** 2021-02-22

**Authors:** Shuhai Jia, Jun Peng, Jiaming Bian, Shuo Zhang, Shunjian Xu, Bao Zhang

**Affiliations:** 1 School of Mechanical and Electrical Engineering, Xinyu University, Xinyu 338004, China; shjia@xjtu.edu.cn (S.J.); xushunjian@126.com (S.X.); zcgyrsrq@126.com (B.Z.); 2 School of Mechanical Engineering, Xi’an Jiaotong University, Xi’an 710049, China; bjm123@stu.xjtu.edu.cn (J.B.); zhangshuo0208@stu.xjtu.edu.cn (S.Z.)

**Keywords:** MEMS, electromagnetic driving, swing actuator, optical switch

## Abstract

A microelectromechanical systems system (MEMS) electromagnetic swing-type actuator is proposed for an optical fiber switch in this paper. The actuator has a compact size of 5.1 × 5.1 × 5.3 mm^3^, consisting of two stators, a swing disc (rotator), a rotating shaft, and protective covers. Multi-winding stators and a multipole rotator were adopted to increase the output torque of the actuator. The actuator’s working principle and magnetic circuit were analyzed. The calculation results show that the actuator’s output torque is decisive to the air gap’s magnetic flux density between the stators and the swing disc. NiFe alloy magnetic cores were embedded into each winding center to increase the magnetic flux density. A special manufacturing process was developed for fabricating the stator windings on the ferrite substrate. Six copper windings and NiFe magnetic cores were electroplated onto the ferrite substrates. The corresponding six magnetic poles were configured to the SmCo permanent magnet on the swing disc. A magnetizing device with a particular size was designed and fabricated to magnetize the permanent magnet of the swing disc. The actuator prototype was fabricated, and the performance was tested. The results show that the actuator has a large output torque (40 μNm), fast response (5 ms), and a large swing angle (22°).

## 1. Introduction

Optical fiber has many advantages compared with electric cables, such as high bandwidth, low loss, a compact size, immunity to electromagnetic interference, and large capacity [1,2,3]. To satisfy the unprecedented demand for an ever-higher fiber network capacity as data network traffic grows, this multiplexing technology is used to enhance communication link capacity. The optical switch is an indispensable component in such a multiplexing system, as it enables the routing of optical data signals without a conversion to electrical signals, and it is independent of data rate and data protocol [4]. A very important function of optical switches is the provisioning of light paths. Optical switches can be categorized into three types according to the switching principles, namely optomechanical switches, interferometric switches, and digital optical switches [5]. Optomechanical switches are widely used in applications with relatively low switching speed. In optomechanical switches, the switching action is performed by some mechanical means, such as prisms, mirrors, and directional couplers, etc. The microelectromechanical systems system (MEMS) optical switch is an important member of optomechanical switches. Compared to other optomechanical switches, a MEMS optical switch has advantages of a compact size, fast switch speed, low insertion loss, low crosstalk, and polarization sensitivity, and it is considered the mainstream in large-capacity switched optical networks [4,6]. In MEMS switches, the light beam is redirected to a desired port by the microactuator.

MEMS devices have both electrical and mechanical components, and they contain at least one movable structure for some mechanical action [7]. Movability is perhaps the most important character of a MEMS, and so the actuator is one of the most important parts of the MEMS. The structure design and driving principles are the two key points for actuators. So far, various driving modes have been studied, such as thermal expansion [8,9], electrostatic force [10,11,12], piezoelectric effect [13,14], shape memory alloys [15,16], and electromagnetic force driving [17,18,19], and a hybrid of thermal and electromagnetic methods [20]. Additionally, metamaterials were designed as the driving units in the MEMS actuators in recent years [21,22,23]. The motion mechanism is mostly based on electrostatic and electromagnetic driving. Actuators based on electrostatic force can be batch-manufactured like integrated circuits, and thus they are one of the most popular drivers. However, the driving force is small and the driving voltage is too high to be compatible with other circuits. Piezoelectric ceramic is widely used in a MEMS for precision driving, but it cannot achieve large displacement motion. Electromagnetic driving has the merits of a large driving force, higher efficiency and longer operation lifetime, and large output displacement.

In this paper, a novel swing-type MEMS actuator is proposed for optical switches. This actuator has an axial magnetic flux structure with multiple windings and magnetic poles. It has the advantages of a large output torque, fast response, a compact size, and good position repeatability.

## 2. Design of Actuator

### 2.1. Structure Design

**Figure 1:**
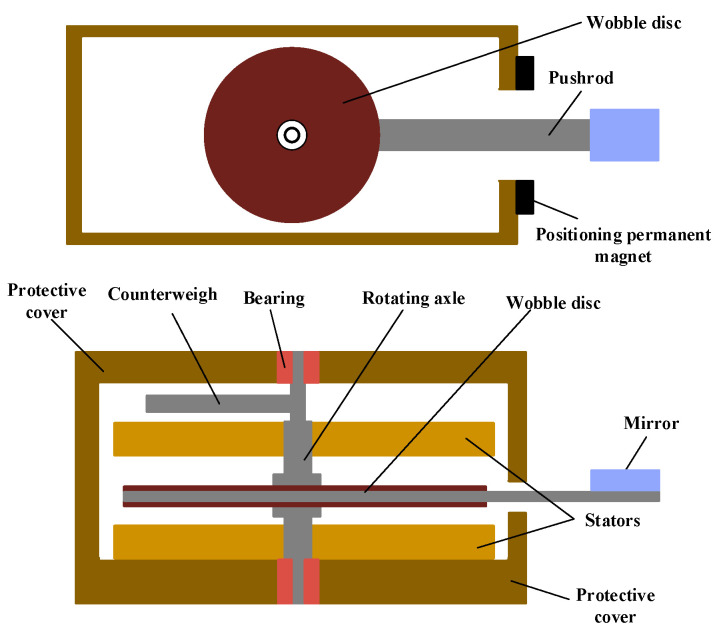
The structure diagram of the swing-type electromagnetic actuator.

In the MEMS optical switch, the actuator is the key element to control the mirrors to move in and out of the light path. To increase the output torque and the response speed of the actuator, an axial magnetic flux structure with multiple windings and magnetic poles is proposed, as shown in Figure 1. The actuator consists of four parts: the stators composed of ferrite substrate and Cu windings; the swing disc made up of a SmCo permanent magnet and silica steel plate; the rotating shaft parts, including a counterweight, rotating shaft, and ruby bearings; and a protective cover and the positioning permanent magnet. The actuator is a three-layer structure, where the swing disc is sandwiched between the two stators. This laminated structure can improve the output torque of the actuator. The magnetic attraction of the upper and lower stators to the disc can be counteracted with each other, which can reduce the friction of the swing disc. The ruby bearings are used in the actuator to alleviate the shaft abrasion, which will decrease the driving voltage and power consumption, and thus prolong the service life. The stator winding can be fabricated by the microprocessing technique.

### 2.2. Working Principle

**Figure 2:**
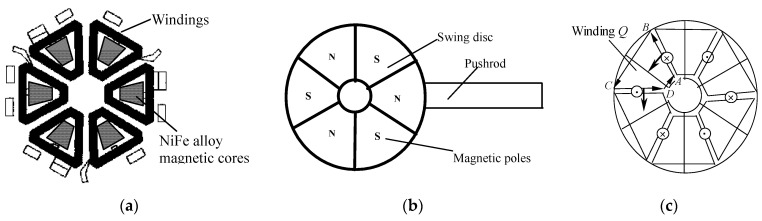
Decomposition diagram of actuator: (**a**) stator windings, (**b**) swing disc, (**c**) interaction between the stator and swing disc.

Figure 2 shows the diagram of the stator and swing disc structure, as well as the interaction between the stator and swing disc. There are six windings on the stator substrate, and a piece of FeNi alloy is embedded into each winding as the magnetic core, as shown in Figure 2a. This magnetic core can reduce the magnetoresistance and increase the output torque. The structure of the swing disc is illustrated in Figure 2b. Six magnetic poles are written in the SmCo permanent magnet of the swing disc. There is a silica steel pushrod at one side of the disc to output displacement. A mirror is mounted on the end of the steel pushrod to control the connection and disconnection of the light path. There is an angle of 30 degrees between the magnetic pole boundary and the winding edge. The magnetizing direction of the permanent material in the swing disc is parallel to the rotating axis. When the current flows through the winding, an ampere force along the circumference of the stator is generated. As shown in Figure 2c, when the current flows through the winding *Q* in the anticlockwise direction (A → B → C → D → A), the radial wires (AB and CD) will be acted on by the anticlockwise force along the circumference of the stator. Because the windings are fixed on the stator substrate, the swing disc will be acted on by the counterforce, which induces the clockwise rotation of the swing disc. When all the windings are energized, the driving force acting on the swing disc is the resultant force of all windings.

When the direction of the electric current in the windings is reversed, the swing disc will be acted on by the inverted force, and the swing disc turns back. Because of the position limitation of the pushrod on the side of the disc and the design of the windings and magnetic poles, the swing disc can only swing in a definite angle range. Two permanent magnets are placed on both sides of the pushrod to maintain the position when the electrical source is disconnected. To reduce the vibration induced by the pushrod, a counterweight is fixed on the rotating axle to balance the load of swing disc.

### 2.3. Analysis of Magnetic Circuit

**Figure 3:**
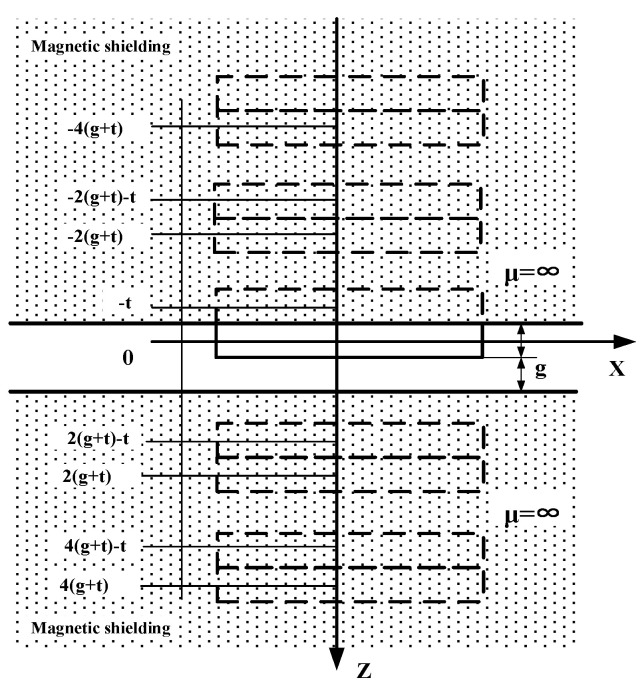
Calculation model of the magnetic field for the swing actuator.

The calculation of the magnetic circuit of the actuator is a complex nonlinear problem. There are various methods of air-gap field calculation, including finite element analysis, equivalent magnetic charge, and Fourier transform. Here, we adopted the Fourier transform to solve the magnetic field in the air gap. The calculation model of the magnetic field for the swing actuator is described in Figure 3 under the following assumptions:

- The magnetic shielding on the upper and lower stators of the swing disc are soft magnetic materials with high permeability, and so its permeability can be approximately considered infinite.
- The distribution of the shielding layer of the swing disc is an infinite space because the air gap between the swing disc and the stator is far smaller than the diameter of the disc.
- The magnetization vector of the swing disc is stable and perpendicular to the surface of the disc, and the magnetization direction of the adjacent magnetic poles is opposite.

According to the calculation model, the magnetic field in the air gap is the sum of the field intensity produced by the disk and its mirror image. The magnetic field intensity on the plane Z can be calculated by Equation (1).

(1) $$\begin{array}{cc} H_{g}(x,y,z)= & \sum_{k=0}^{\infty }[H_{d}\left(x,y,\left(z-2\left(k+1\right)(g+t)\right)\right)+H_{d}\left(x,y,\left(z+t-2\left(k+1\right)(g+t)\right)\right)\\ & +H_{d+}\left(x,y,\left(z+2k(g+t)\right)\right)+H_{d}\left(x,y,\left(z+t+2kd\right)\right)],\left(t/2<z<g+t/2\right) \end{array}$$

where ${\vec{H}}_{d}$ is the field intensity of swing disc in the free space, *t* is the thickness of the swing disc, and *g* is the distance between the disc surface and the soft magnet.

The magnetic flux density of the disc can be acquired by Equation (2).

(2) $$\vec{B}=\mu_{0}(\vec{H}+\vec{M})$$

**Figure 4:**
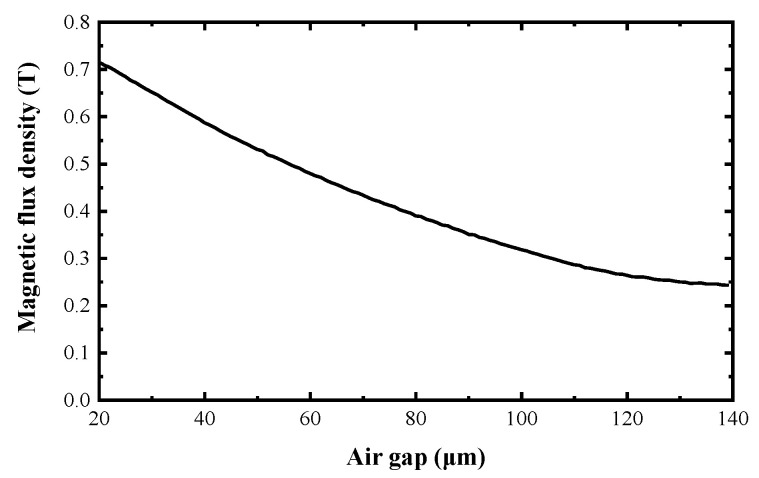
Relation between the magnetic flux density and air gap dimension.

For the magnetic flux density in the air gap, the magnetization vector $\vec{M}$ is equal to 0. The relation between the magnetic flux density and the dimension of the air gap is illustrated in Figure 4.

The windings of the actuator can be regarded as an integration of many wire units. When the current *i* flows through the wire with a length of Δ*l*, the electromagnetic force can be calculated by Equation (3). The sum of the torque acted on the swing disc can be obtained from Equation (4):

(3) $$\Delta \vec{F}=i\Delta \vec{l}\times \vec{B}$$

(4) $$\vec{T}=\sum_{i}\Delta T=\sum_{i}i\left|{\vec{r}}_{i}\right|\times \left|\Delta {\vec{l}}_{i}\right|\times B_{i}\cos\theta_{i}$$

where $\vec{T}$ is the sum of the torque, *θ* is the angle between the current and the direction of magnetic flux, and *r* is the radius of rotation.

**Figure 5:**
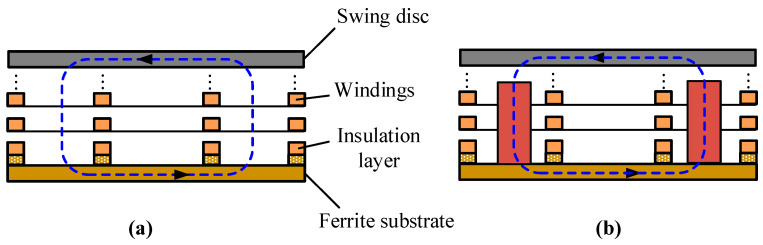
Magnetic circuit of the actuator. (**a**) Nonembedded NiFe alloy. (**b**) Embedded NiFe alloy.

From Equation (4), it can be observed that the output torque *T* is directly proportional to the magnetic flux density *B*, the electric current *i*, the wire length *l,* and the rotation radius *r*. In consideration of the compact size of actuator, the optimal measure to enhance the output torque is to increase the magnetic flux density. From Figure 4, it can be seen that the magnetic flux density in the air gap attenuates with the increase of the air gap. The air gap cannot be too small; otherwise it could affect the assembly of actuator. NiFe alloy with high permeability was used to increase the magnetic flux density in the air gap. The NiFe alloy magnet is embedded into the windings as a magnetic core as shown in Figure 5.

## 3. Fabrication of Actuator

The fabrication of an actuator is a manufacturing process that integrates bulk micromachining, precision manufacturing, and assembly. The fabrication of the stator and the magnetization of swing disc are the key procedures of the fabrication.

### 3.1. Stator Fabrication

#### 3.1.1. Material Preparation

The substrate of the stators is the supporting body of the wings, and the magnetic field lines can form a closed loop. The material of the substrate must satisfy three requirements: machinability, good magnetic conductivity, and nonconductivity. Here, the ferrite was chosen as the material of the substrate. The NiFe alloy was selected as the magnetic core in the windings due to its high permeability and good conductivity. The thickness of the single layer of NiFe alloy is about 20 μm. Mask electroplating was used to form the NiFe alloy film. The electroplating solution is NiSO_4_+NiCl_2_+FeSO_4_, and its pH is in the range of 5–6. Al_2_O_3_ was used as the insulating material of the interface layer of windings because of its good insulation, temperature stability, and durability, which can effectively solve the aging problem of the insulation material and increase the lifetime of the actuator.

#### 3.1.2. Manufacturing Process

**Figure 6:**
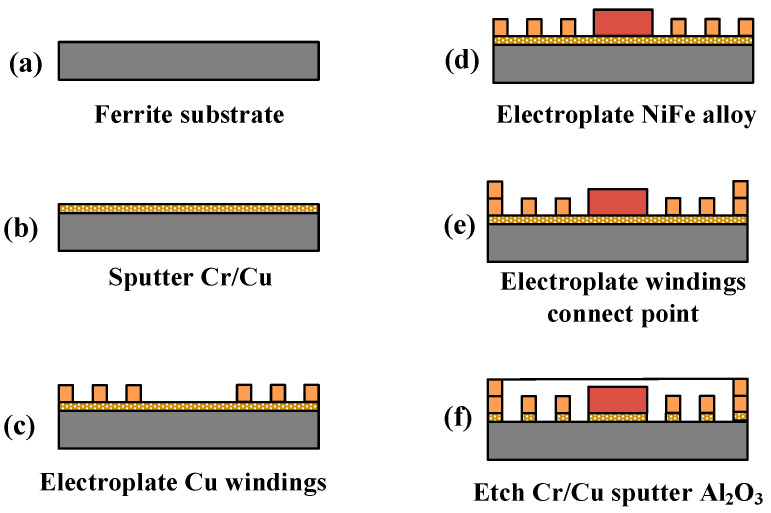
Manufacturing process of the stators.

Generally, micromachining technology is mainly carried out on silicon materials. A special processing technology was developed for the ferrite, which includes masking, photoetching, sputtering, and dry etching, as shown in Figure 6. The procedures of fabrication are concluded as follows:

- One seed layer composed of Cr/Cu was sputtered onto the cleaned ferrite substrate, as illustrated in Figure 6b.
- The photoresist AZ4620 was coated onto the surface of the seed layer, and then we photoetched the mask window for electroplating the copper windings. The electroplating speed of the copper windings was 50 nm/min. After copper electroplating, the photoresist was removed, as shown in Figure 6c.
- In the same way as in Figure 6b, NiFe was electroplated in the center of the windings at a speed of 1 μm/min, as shown in Figure 6d. The electroplating process was conducted at a temperature of 40 ± 2 °C. The coercivity and relative permeability of the NiFe film was controlled at about 8–16A/M and 8000–9000.
- The coat photoresist, the photoetch mask window, and the electroplate of the connecting point of two layers of windings were formed, as shown in Figure 6e.
- Removal of the photoresist and etch of the seed layer by sputtering was performed. The insulation layer Al_2_O_3_ was sputtered on the substrate and then the uneven area was ground and polished, as shown in Figure 6f. The thickness of each Al_2_O_3_ layer is about 25–26 μm, and the maximum speed of sputtering is 25–26 μm/h.
- The remaining four layers of winding were fabricated by similar process steps.

### 3.2. Swing Disc Fabrication

The swing disc is one of the key components of the actuator, and it determines the magnitude of output toque. It is important to select the optimal material for the permanent magnet in the disc. Taking the performance of manufacturing and pole writing into consideration, two SmCo permanent magnets were glued onto the upper and lower surfaces of the swing disc made up of silicon steel. The permanent magnetic material has a property of magnetic remanence *B_r_* (0.8–0.9 T), a minimum intrinsic coercivity *H_c_* (1592 kA/m), and a magnetic energy product (*BH*)*_max_* (119–143 kJ/m^3^). The swing disc was manufactured by electrodischarge machining.

**Figure 7:**
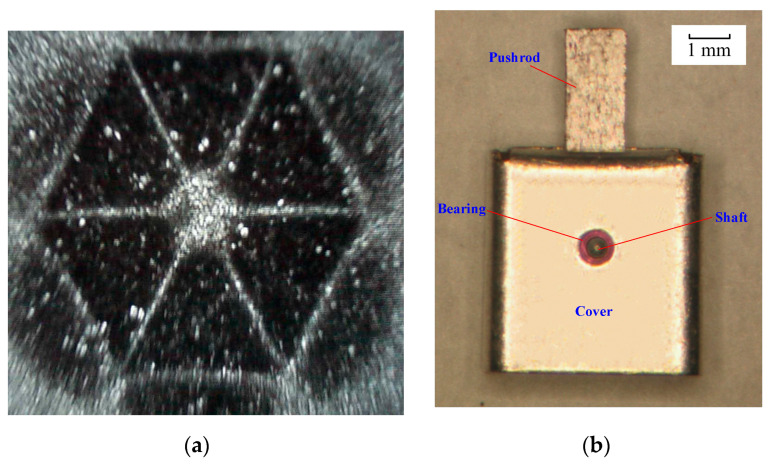
(**a**) Magnetic photograph of magnetic poles. (**b**) Micrograph of the proposed microelectromechanical systems system (MEMS) swing-type actuator.

The magnetic poles writing is also a key process for the actuator. The uniformity and magnetic saturation of the poles are decisive to the performance of the swing disc. A magnetizing device with a particular size was designed and fabricated to magnetize the permanent magnet of the swing disc. The magnetic photograph of the magnetic poles is shown in Figure 7a. The picture shows that the magnetic poles are uniform in size and distribution. The magnetic remanence of the swing disc was measured. The results show that the magnetic remanence in the swing disc is the same as that of the permanent magnet block, which indicates that the swing disc is in the magnetic saturation state. The actuator porotype with a compact size of 5.1 × 5.1 × 5.3 mm^3^ is fabricated as shown in Figure 7b.

## 4. Performance Testing

**Figure 8:**
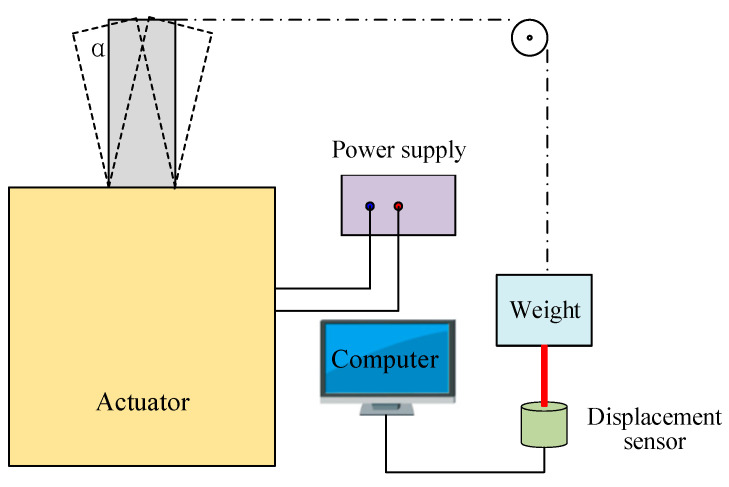
Diagram of test platform for the measurement of the output torque and swinging angle.

The output torque, swinging angle, and switching time were the key parameters for the optical switches. A loading test platform was established to measure the output torque and swinging angle, as shown in Figure 8. A weight was connected to the pushrod by a string, and a pulley was used to support and guide the string. The proposed actuator was controlled by a signal generator that drags up the weight. Simultaneously, a distance sensor was adopted to detect the displacement of weight. The maximum output torque can be obtained by the product of the maximum weight and the push rod length. The length of the pushrod is 8 mm, and the maximum weight is 0.5 g, which indicates that the maximum output torque is 40 μNm.

**Figure 9:**
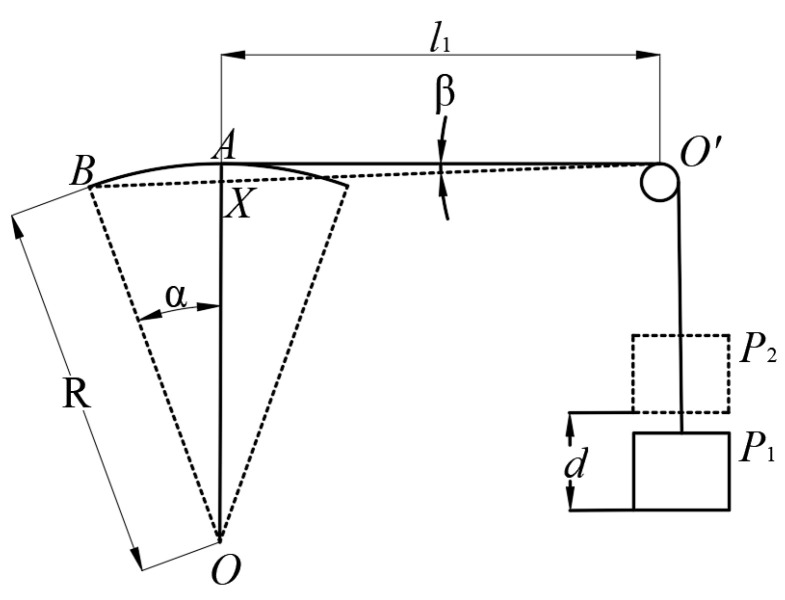
Calculation model for swing angle.

The calculation model of the swinging angle is shown in Figure 9. The swing angle can be calculated by Equation (5).

(5) $$\sin\alpha =\frac{\bar{BX}}{\bar{OB}}=\frac{\bar{BO^{\prime }}-\bar{XO^{\prime }}}{\bar{OB}}=\frac{l_{1}+d-l_{1}/\sin\beta }{R}$$

where *α* is the swing angle, *d* is displacement of weight detected by the sensor, and *R* is the swing radius of pushrod.

Because the angle *β* is far too small and sin*β* is approximately 1, the swing angle is equal to arcsin(*d/R*). The testing results show that and the actuator has a maximum swing angle of 22° and a swing displacement of 5.4 mm.

**Figure 10:**
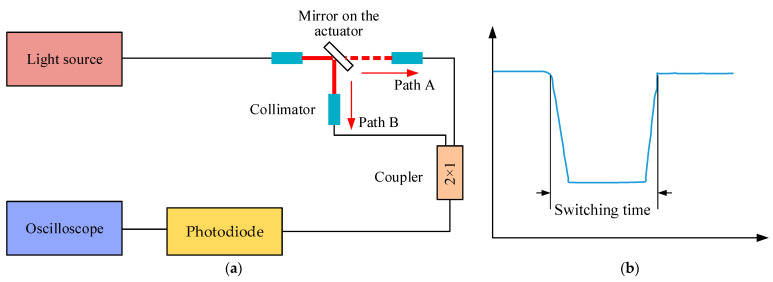
(**a**) diagram of the test platform for the measurement of switching time. (**b**) definition of switching time.

**Figure 11:**
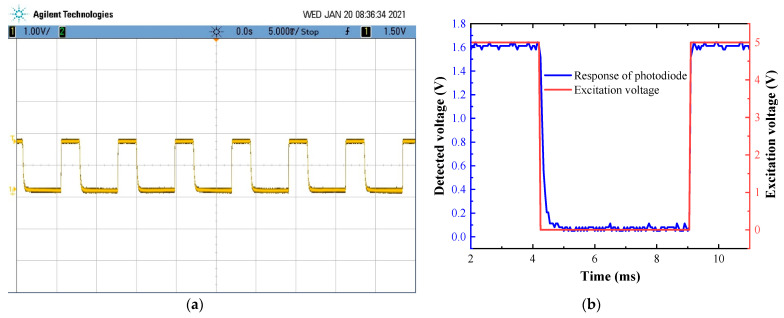
(**a**) Oscilloscope display graphics and (**b**) response of photodiode and excitation voltage.

Previously, the mechanical parameters of the proposed actuator were tested. The key functional parameter (switching time) was tested, and the experimental setup is shown in Figure 10a. The light from the laser was divided into two paths by the reflected mirror in the actuator. The two beams of light were coupled into one by a fiber coupler, and then the coupled light was detected using a photodetector, which was composed of a photodiode and amplifier. The output of the photodetector was illustrated on the oscilloscope (MSO7032A, Agilent Technologies, Santa Clara, CA, USA). When the pushrod of the actuator is in the position shown in Figure 9, the light is reflected by the mirror and travels through path B. The light travels through path A when the mirror swings away from this position. With the reciprocating swing of the actuator, the amplitude of output signal shows a downward and upward trend. The switching time can be obtained from the time interval between the first decay to 10% of the stable amplitude and the next rise to 90% of the stable amplitude, as shown in Figure 10b. The testing result is illustrated in Figure 11. The minimum switching time is 5 ms, which is comparable to the commercial products (≤30 ms [24], ≤15 ms [25], 0.5–1 ms [26]).

## 5. Conclusions

An electromagnetic MEMS swing actuator was developed in this paper. The actuator has a sandwich structure, including two stators, a swing disc, a rotating shaft, and covers.

The working principle and the magnetic circuit of the proposed actuator were analyzed. The results show that the actuator’s output torque is decisive to the air gap’s magnetic flux density between the stators and swing disc. An optimal measure was taken to increase the output torque using NiFe alloy magnetic cores that were embedded into each winding center. Six copper windings and NiFe magnetic cores were electroplated on the ferrite substrates. The corresponding six magnetic poles were distributed onto the SmCo permanent magnet of the swing disc.

The actuator prototype was fabricated, and experiment platforms were established to test the performance of the actuator. The design and manufacturing process enabled the proposed actuator to have a large output torque of 40 μNm, a fast response of 5 ms, and a large swing angle of 22°. This actuator can be used for the optical fiber switches and the automatic switching of the light path in other applications.
